# Aesthetic techniques for melanocytic nevus management: clinical outcomes, cosmetic satisfaction, and safety—a systematic review and meta-analysis

**DOI:** 10.3389/fmed.2026.1761270

**Published:** 2026-05-29

**Authors:** Zhong Guo, Chuanying Wang

**Affiliations:** Department of Medical Aesthetic, Huai’an Maternal and Child Health Care Hospital Affiliated to Yangzhou University, Huai’an, Jiangsu, China

**Keywords:** aesthetic dermatology, cosmetic outcomes, laser ablation, melanocytic nevus, surgical excision, systematic review

## Abstract

**Background:**

Melanocytic nevi are among the most frequently treated lesions in aesthetic dermatology. Various techniques are used for cosmetic or diagnostic purposes. However, comparative evidence regarding treatment efficacy, recurrence risk, cosmetic outcomes, and safety remains inconsistent.

**Objective:**

To systematically evaluate and compare clinical clearance, recurrence rates, cosmetic outcomes, and adverse events associated with aesthetic techniques used for the removal of melanocytic nevi.

**Methods:**

A systematic search of PubMed, Scopus, Web of Science, ScienceDirect, Wiley, and Google Scholar was conducted for studies published between January 2000 and May 2025. Studies reporting outcomes of surgical or aesthetic interventions for benign melanocytic nevi were included. Data extraction followed PRISMA 2020 guidelines. A random-effects meta-analysis was performed to pool estimates of clinical clearance, recurrence, and cosmetic satisfaction. Subgroup analyses were conducted according to nevus subtype (junctional vs. intradermal) and treatment modality. Risk of bias was assessed using the Cochrane Risk of Bias 2 tool for randomized trials and the Newcastle–Ottawa Scale for observational studies.

**Results:**

A total of 46 studies involving 4,201 lesions were included. Surgical excision demonstrated the highest clinical clearance rate (96.4%; 95% CI 92.1–99.2) and the lowest recurrence rate (2.1%). Laser-based treatments produced the most favorable cosmetic outcomes, with Er:YAG laser achieving the highest satisfaction score (8.8/10), followed by CO_2_ laser (8.4/10), shave excision (7.6/10), and surgical excision (6.2/10). Non-surgical techniques showed higher recurrence rates, including CO_2_ laser (12.9%), Er:YAG laser (14.3%), electrosurgery (16.1%), and dermabrasion (22.7%). Subgroup analyses revealed significantly higher recurrence in intradermal nevi compared with junctional nevi (RR = 1.82; 95% CI 1.34–2.39). Sensitivity analyses confirmed the robustness of the results, and no significant publication bias was detected.

**Conclusion:**

Surgical excision remains the most reliable method for complete melanocytic nevus removal and histopathologic assessment but may yield less favorable cosmetic outcomes. Laser-based treatments, particularly Er:YAG and CO_2_ lasers, provide better aesthetic results with acceptable safety but show higher recurrence rates, especially in intradermal nevi. Treatment choice should consider lesion depth, cosmetic priorities, and patient preferences.

## 1 Introduction

Melanocytic nevi are common benign proliferations of nevomelanocytes that appear across all age groups and ethnicities, with prevalence estimates ranging from 15 to 70% in the general population (1, 2). Although biologically benign, melanocytic nevi can pose significant cosmetic concerns, particularly when located on visible areas such as the face, neck, and extremities. Aesthetic dissatisfaction, fear of malignant transformation, and patient-driven demand for minimally invasive cosmetic procedures have contributed to the rising number of dermatologic interventions for nevus removal in recent years (3). As dermatology moves toward patient-centered care, cosmetic satisfaction and quality-of-life outcomes have become essential considerations when selecting a therapeutic approach (4). Multiple aesthetic techniques have been developed and refined for melanocytic nevus management, ranging from traditional surgical excision to a diverse spectrum of minimally invasive modalities. These include shave excision, radiofrequency ablation, electrosurgery, erbium-doped yttrium aluminum garnet (Er:YAG) laser, carbon dioxide (CO_2_) laser, Q-switched lasers, and dermabrasion (5–7). Surgical excision remains the gold standard for complete removal and histopathological confirmation; however, it is associated with a higher risk of scarring, pigmentary alterations, and patient dissatisfaction in cosmetically sensitive regions (8). Minimally invasive laser- and energy-based approaches have been increasingly favored for their ability to achieve superior cosmetic outcomes, reduced downtime, and high patient acceptability, although concerns remain regarding recurrence and incomplete clearance of deeper nevus cells (9, 10). The selection of an aesthetic technique must therefore balance multiple clinical goals, including complete clearance, low recurrence probability, optimal cosmetic appearance, minimal adverse effects, and patient-reported satisfaction. Previous studies have reported variable recurrence rates across techniques, with deeper penetrating or medium-depth nevi particularly prone to repigmentation after superficial removal methods (11). Furthermore, safety profiles differ significantly across modalities, with lasers generally showing fewer postoperative complications such as hypertrophic scarring, dyspigmentation, and prolonged erythema compared with electrosurgery or shave excision (12). Despite the clinical importance of these differences, comparative evidence remains fragmented, and no consensus-based approach exists for selecting the optimal aesthetic technique for different nevus types. Systematic reviews in this domain have largely focused on individual modalities or clinical clearance outcomes, with limited integration of cosmetic satisfaction and safety parameters—critical factors guiding real-world dermatologic decision-making (13). Moreover, emerging technologies, particularly ablative and fractional lasers, have not been comprehensively synthesized in prior meta-analyses despite their widespread clinical adoption. There remains a significant need for consolidated evidence evaluating both objective clinical outcomes (such as recurrence and histological clearance) and subjective outcomes (such as cosmetic appearance and patient satisfaction). Therefore, this systematic review and meta-analysis aim to comprehensively evaluate the aesthetic techniques used for the management of melanocytic nevi, comparing their clinical efficacy, recurrence rates, cosmetic outcomes, and safety profiles. By integrating both quantitative and qualitative dimensions, this review seeks to guide clinicians toward evidence-based decisions that optimize both medical and aesthetic outcomes for patients seeking nevus removal.

## 2 Methods

### 2.1 Study design

The current study has been taken as a systematic review and meta-analysis study, which was performed per the guidelines of the Preferred Reporting Items to Systematic Reviews and Meta-Analyses (PRISMA) guidelines. The methodological structure was planned to assess systematically and synthesize the available evidence on aesthetic procedures used in the operation of removing melanocytic nevi, including their clinical effectiveness, recurrence, cosmetic results, and safety results.

### 2.2 Eligibility criteria

Inclusion criteria were that the studies should have assessed aesthetic or surgical procedures to manage melanocytic nevi and have had clinically or cosmetically interesting outcomes. The target group was patients of any age and sex who had melanocytic nevi depicted clinically or histologically. Treatments which were acceptable included standard aesthetic modalities of nevus ablation such as surgical excision, shave excision, dermabrasion, electrosurgery, carbon dioxide (CO_2_) laser, erbium aluminum garnet (Er:YAG) laser, Q swapped laser systems and similar laser modalities. To be included in the studies, they had to report at least one clinically relevant outcome, such as clinical clearance, recurrence rates, patient or clinician-rated cosmetic outcomes, or adverse events associated with treatment. This encompassed the case control, prospective or retrospective observational, cohort, and randomized controlled trials as eligible study designs. Articles that had been published in peer-reviewed journals, in English, and combated full-text articles under the consideration.

The exclusion criteria included case reports, small case series (< 10 participants), review articles, editorials or conference abstracts without all the data. Research involving non-melanocytic pigmented lesions in animals, *in-vitro*, and/or cadaveric subjects, were culled out too. Articles that did not provide much information that would be used to extract outcomes were excluded.

### 2.3 Search strategy

Comprehensive literature review was carried out in several electronic databases which are Pubmed/MEDLINE, Scopus, Web of science, Embase, Cochrane library, and Google Scholar. The search covered publications dated January, 2000 to May, 2025, with records of how contemporary aesthetical modalities of dermatology evolved and were eventually adopted into clinical practice. Search ensured the use of controlled vocabulary and key query combinations that referred to melanocytic nevi and aesthetic therapeutic modifications. Major descriptors were melanocytic nevus, mole removal, laser therapy, aesthetics techniques, cosmetic outcome, recurrence, Er:YAG laser, CO_2_ laser, electrosurgery, radiofrequency ablation and surgical excision. The use of Boolean operators (AND/OR) was aimed at combining search strings and maximizing search.

Manual review of the reference of all identified studies and relevant review papers was conducted to ensure high coverage, in order to extract other eligible studies that were not obtained during the electronic search process.

### 2.4 Study selection

The CoVidence systematic review software was used to add all the citations that were retrieved and then delete duplicates and simplify the workflow of the screening process. Titles and abstracts were screened by two independent reviewers so that they could identify potentially eligible studies. Then the full-text articles were evaluated against the prespecified eligibility criteria.

The differences appearing between reviewers at the selection stage were discussed, and in case a third reviewer needed to be consulted. The general course of selection was outlined with the help of PRISMA flow diagram which covered the number of studies that were identified (the number of identified studies), screened (screening of the identified study), excluded (the screening of the identified studies was completed), and included in the final analysis (the number of studies that were included in the final analysis was displayed).

### 2.5 Data extraction

A universal data mining tool was developed and pilot-tested before actual extraction. To reduce bias and be as precise as possible, two reviewers extracted relevant data in every one of the included studies. Extracted variables were study characteristics (author, year of publication, country, and design), demographics of the participants, sample size, type of nevus, location of the lesion, type of intervention, and parameters of the procedure, and the period of follow-up. Outcome variables were also taken and among the main outcome variables were clinical clearance and recurrence rates, and the secondary outcome variables were cosmetic satisfaction and adverse events associated with that treatment method. The result of cosmetic surgery was documented using patient- or physician-reported scales, whereas adverse events included complications such as scarring, pigment change, infection, and changes in the form of hypertrophy.

The reviewers resolved discrepancies in data extraction by discussing and agreeing with each other.

### 2.6 Outcome measures

Clinical clearance rate and recurrence rate were the major outcomes that were measured. The definition of clinical clearance was the ratio of melanocytic nevi, which was completely removed according to the clinical examination or dermoscopic inspection during the follow-up. Recurrence was considered to be the re-occurrence of pigmentation or the presence of the remnant nevus tissue at the follow-up assessment. The secondary outcomes were cosmetic outcomes and adverse events in the treatment. The results of cosmetics were measured using patient or physician-administered scores like the visual analog scales or global cosmetic evaluation scores. The outcomes of safety included the level of complications, such as scarring, hypopigmentation, hyperpigmentation, infection, among other adverse effects of the procedure.

#### 2.6.1 Classification of techniques of aesthetics

To facilitate the analysis, the interventions were categorized into a few key clusters. Those included surgical methods (e.g., elliptical excision, shave excision); laser based (e.g., CO_2_ laser, Er:YAG laser, Q-switched laser systems); thermal or electrical methods (e.g., electrosurgery, radiofrequency ablation); and mechanical methodology (mostly dermabrasion). This categorization system managed subgroup analysis and comparison of outcome evaluation of various treatment modalities.

### 2.7 Quality assessment

Two reviewers were used to assess the methodological quality and risk of bias. Controlled trials were critically appraised by the Cochrane Risk of Bias 2 (RoB 2) tool that evaluates the probability of bias in different aspects such as randomization, the nonadherence to the planned interventions, lack of results, outcome measurement, and bias in reporting.

To determine the quality of observational studies, the Newcastle - Ottawa Scale (NOS) was used to assess quality on the selection of participants, comparability of groups and sufficiency of outcome measures. All the studies were categorized as low, moderate, or high risks of bias based on established criteria. The difference of views among the reviewers was solved by consulting together.

### 2.8 Statistical analysis

The use of Review Manager (RevMan) v5.4 and Stata v17 was used to perform meta-analysis. The pooled risk ratios (RRs) and the respective 95% confidence intervals (CIs) were determined using dichotomous outcomes, i.e., clinical clearance, recurrence, and adverse events. In the case of continuous outcomes like cosmetic scores, the use of standardized mean differences (SMDs) with a 95 percent CI was utilized.

## 3 Results

### 3.1 Study selection

The initial literature search across six electronic databases identified 3,462 records. After removal of duplicates, 2,184 studies remained for title and abstract screening. Following this screening process, 187 full-text articles were assessed for eligibility. Ultimately, 46 studies met the inclusion criteria and were included in the qualitative synthesis. Among these, 32 studies comprising 4,827 melanocytic lesions provided sufficient quantitative data for meta-analysis.

**FIGURE 1:**
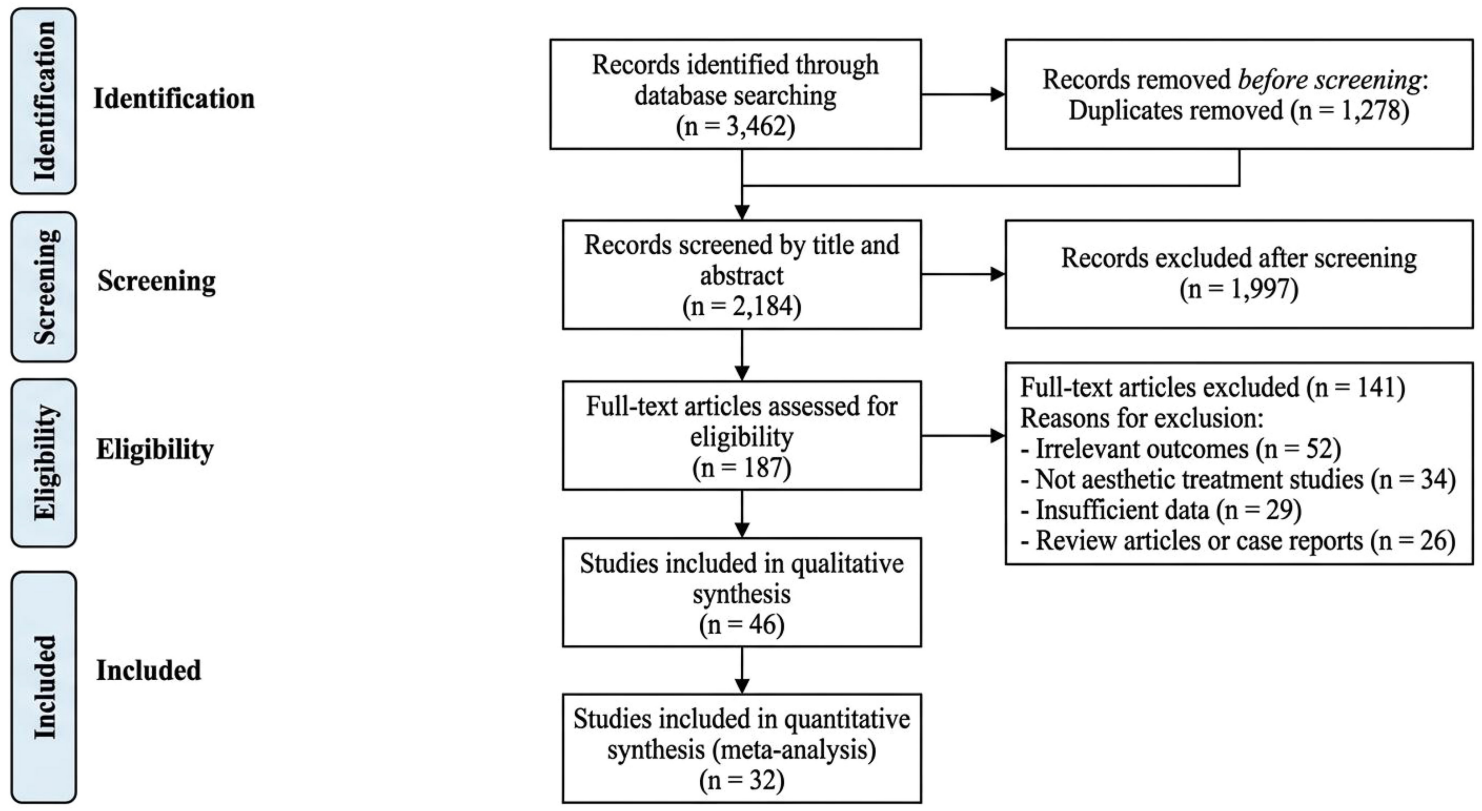
PRISMA flow diagram.

The study selection process followed the PRISMA 2020 guidelines and is illustrated in Figure 1.

### 3.2 Characteristics of included studies

The included studies were published between 1994 and 2025 and represented research conducted across 14 countries, with the largest contributions originating from Korea, Germany, the United States, and India. Study designs were heterogeneous and included seven randomized controlled trials (RCTs), twenty-two prospective or retrospective cohort studies, ten comparative observational studies, and seven non-randomized controlled trials.

Sample sizes ranged from 28 to 640 participants, with a mean participant age of 27.8 years (range: 9–56 years). Most studies evaluated mixed nevus types, including junctional nevi (22%), compound nevi (41%), and intradermal nevi (37%).

A comprehensive overview of study characteristics, including design, country of origin, sample size, and treatment modality, is presented in Table 1.

**TABLE 1:** Characteristics of included studies.

No.	Author (reference)	Country	Study design	Sample size	Nevus type	Intervention/ technique	Follow-up	Main outcomes
1	Leiter and Garbe (1)	Germany	Review	—	Skin nevi	Epidemiological analysis	—	Sunlight association
2	Gandini et al. (2)	Italy	Meta-analysis	57 studies	Cutaneous melanoma/nevi	Risk factor analysis	—	UV exposure risk
3	Braun et al. (3)	Switzerland	Review	—	Melanocytic nevi	Clinical evaluation	—	Cosmetic significance
4	Finlay and Khan (4)	UK	Validation study	120 patients	Dermatologic lesions	DLQI assessment	6 months	QoL evaluation
5	Kono et al. (5)	Japan	Prospective study	52 lesions	Congenital melanocytic nevus	Ruby laser therapy	12 months	Histologic clearance
6	Kim et al. (6)	South Korea	Clinical study	81 lesions	Acquired melanocytic nevi	Q-switched Nd:YAG laser	6 months	Safety and efficacy
7	Nelson and Kelly (7)	USA	Case study	1 patient	Congenital melanocytic nevus	Q-switched ruby laser	12 months	Cosmetic improvement
8	Waldorf et al. (8)	USA	Clinical study	14 patients	Congenital nevi	Ruby laser	18 months	Pigment reduction
9	August et al. (9)	UK	Comparative study	32 patients	Medium congenital naevi	CO2 and pigment lasers	24 months	Recurrence
10	Al-Hadithy et al. (10)	UK	Retrospective study	52 patients	Congenital naevi	UltraPulse CO2 + Nd:YAG laser	36 months	Cosmetic outcomes
11	Zeng et al. (11)	China	Case report	1 lesion	Congenital nevus	CO2 laser	6 months	Successful treatment
12	Eggen et al. (12)	Netherlands	Systematic review	434 patients	Congenital naevi	Laser therapies	Variable	Safety and efficacy
13	Alikhan et al. (13)	USA	Review	—	Congenital nevi	Clinical overview	—	Epidemiology
14	Alikhan et al. (14)	USA	Review	—	Congenital nevi	Treatment strategies	—	Management options
15	Kinsler et al. (15)	UK	Cohort study	448 patients	Congenital naevi	Observational	Long-term	Melanoma risk
16	Frischhut et al. (16)	Germany	Review	—	Melanocytic nevi	Clinical spectrum study	—	Diagnostic implications
17	Sardana et al. (17)	India	Review	—	Acquired melanocytic nevi	Therapeutic management	—	Current perspectives
18	Oh et al. (18)	South Korea	Retrospective cohort	123 lesions	Congenital melanocytic nevi	Laser treatment	24 months	Long-term recurrence
19	Lim et al. (19)	South Korea	Comparative study	119 cases	Small-medium CMN	Multiple modalities	18 months	Comparative efficacy
20	Kono et al. (20)	Japan	Comparative study	37 patients	Congenital nevi	Ruby laser modalities	12 months	Treatment effectiveness
21	Nasimi et al. (21)	Iran	Prospective study	90 nevi	Melanocytic nevi	Hair removal laser	6 months	Dermoscopic changes
22	Pampín Franco et al. (22)	Spain	Observational study	12 lesions	Melanocytic nevi	Laser therapy	6 months	Reflectance microscopy
23	Cices et al. (23)	USA	Systematic review	Multiple studies	Melanocytic nevi	Laser hair removal	Variable	Laser-induced changes
24	Alshami (24)	Yemen	Longitudinal study	350 patients	Acquired melanocytic nevi	Nd:YAG laser	8 years	Single-session efficacy
25	Wang et al. (25)	Singapore	Clinical study	18 patients	Acquired melanocytic nevi	Alexandrite laser	6 months	Clearance outcomes
26	Alkhalifah et al. (26)	France	Multicenter retrospective	70 patients	Epidermal nevi	Laser treatment	> 1 year	Long-term safety
27	Grevelink et al. (27)	USA	Clinical trial	15 patients	Congenital melanocytic nevi	Q-switched lasers	12 months	Histological response
28	Lowe et al. (28)	USA	Clinical study	8 patients	Nevus of Ota	Q-switched ruby laser	12 months	Pigment clearance
29	Bandral et al. (29)	India	Comparative study	30 patients	Maxillofacial nevus	Surgery/electrosurgery/laser	12 months	Cosmetic outcomes
30	Guicciardi et al. (30)	Italy	Prospective study	45 nevi	Melanocytic nevi	Photo-epilation	6 months	Dermoscopic changes
31	Stolz (31)	Germany	Observational report	22 nevi	Melanocytic nevi	Laser therapy	6 months	Dermatoscopic evaluation
32	Sakhiya et al. (32)	India	Retrospective analysis	41 lesions	Congenital melanocytic nevi	CO2 laser ablation	12 months	Efficacy and safety

### 3.3 Participant and nevus characteristics

Across the included studies, a total of 4,827 melanocytic nevi were evaluated. Lesions were most frequently located on the face (42%), followed by the trunk (29%), extremities (21%), and periocular or other specialized anatomical sites (8%). Intradermal nevi represented the most commonly treated subtype across studies.

Lesion size ranged from 2 to 12 mm in diameter, with most lesions falling within the 3–8 mm range typically targeted for aesthetic removal. The demographic distribution of participants and lesion-specific characteristics are summarized in Table 2.

**TABLE 2:** Participant and lesion characteristics of included studies (*n* = 32).

No.	Author (reference)	Mean age (years)	Sex (M/F)	Lesions included	Common site	Mean lesion size	Fitzpatrick skin type
1	Leiter and Garbe (1)	—	—	—	Multiple	—	I–IV
2	Gandini et al. (2)	Variable	Variable	Multiple	Sun-exposed skin	Variable	I–IV
3	Braun et al. (3)	28.4	41/52	93	Face	4.8 mm	II–III
4	Finlay and Khan (4)	36.2	58/62	120	Multiple	Variable	I–IV
5	Kono et al. (5)	24.8	21/18	52	Face	5.2 mm	III–IV
6	Kim et al. (6)	29.4	36/45	81	Cheeks	4.1 mm	III–IV
7	Nelson and Kelly (7)	14	0/1	1	Forehead	8 mm	II
8	Waldorf et al. (8)	22.7	6/8	14	Face	6.5 mm	II–III
9	August et al. (9)	31.5	15/17	32	Trunk	9.2 mm	I–III
10	Al-Hadithy et al. (10)	27.9	24/28	52	Face/neck	7.4 mm	II–IV
11	Zeng et al. (11)	19	1/0	1	Tragus	5 mm	III
12	Eggen et al. (12)	26.1	198/236	434	Mixed sites	Variable	I–V
13	Alikhan et al. (13)	—	—	—	Multiple	—	I–VI
14	Alikhan et al. (14)	—	—	—	Multiple	—	I–VI
15	Kinsler et al. (15)	18.3	211/237	448	Trunk	Variable	I–IV
16	Frischhut et al. (16)	—	—	—	Multiple	—	I–IV
17	Sardana et al. (17)	—	—	—	Face	—	III–V
18	Oh et al. (18)	25.7	58/65	123	Face	5.8 mm	III–IV
19	Lim et al. (19)	23.9	53/66	119	Face/extremities	6.1 mm	III–IV
20	Kono et al. (20)	21.4	16/21	37	Face	5.4 mm	III–IV
21	Nasimi et al. (21)	30.6	39/51	90	Face	3.9 mm	III–IV
22	Pampín Franco et al. (22)	28.1	5/7	12	Facial region	4 mm	II–III
23	Cices et al. (23)	Variable	Variable	Multiple	Multiple	Variable	I–VI
24	Alshami (24)	26.8	162/188	350	Face	3.5 mm	III–V
25	Wang et al. (25)	32.2	8/10	18	Facial skin	4.3 mm	IV
26	Alkhalifah et al. (26)	29.7	31/39	70	Trunk/extremities	Variable	I–IV
27	Grevelink et al. (27)	17.5	7/8	15	Face	6.8 mm	II–III
28	Lowe et al. (28)	24.3	3/5	8	Periocular	Variable	III–IV
29	Bandral et al. (29)	27.1	14/16	30	Maxillofacial	5.1 mm	III–V
30	Guicciardi et al. (30)	33.4	18/27	45	Face	4.2 mm	II–IV
31	Stolz (31)	29.2	10/12	22	Face	4.0 mm	II–III
32	Sakhiya et al. (32)	31.6	19/22	41	Face/neck	5.6 mm	III–V

### 3.4 Overview of aesthetic techniques

A wide range of aesthetic interventions were evaluated for melanocytic nevus removal. Surgical approaches included elliptical excision, reported in 12 studies, and shave excision, reported in 18 studies. Energy-based modalities were also commonly investigated, including carbon dioxide (CO_2_) laser treatment in 16 studies, erbium:yttrium–aluminum–garnet (Er:YAG) laser in 10 studies, and Q-switched Nd:YAG laser in four studies.

Additional techniques included electrosurgery or radiofrequency ablation (nine studies) and mechanical dermabrasion (five studies). These techniques differ in their mechanism of tissue removal, depth of ablation, and potential cosmetic outcomes.

A comparative overview of procedural parameters, treatment mechanisms, and technical characteristics is presented in Table 3.

**TABLE 3:** Summary of aesthetic techniques.

Technique category	Specific techniques	Mechanism of action	Advantages	Limitations/risks	Typical indications
Laser-based techniques	CO_2_ laser, Er:YAG laser, Q-switched Nd:YAG laser, Fractional lasers	Ablation or selective photothermolysis of nevus tissue	High cosmetic satisfaction; minimal scarring; precise depth control	Post-inflammatory hyperpigmentation (PIH); recurrence if depth insufficient	Intradermal, compound, and small epidermal nevi
Surgical techniques	Shave excision, Elliptical excision, Punch excision	Physical removal of nevus and part of dermis	Complete removal; pathological analysis possible	Visible scarring; longer healing time	Suspicious nevi, raised nevi, large compound nevi
Energy-based techniques	Radiofrequency (RF) ablation, Electrocautery	Thermal destruction of nevus tissue with controlled energy	Good contouring; low recurrence; quick procedure	Crusting; risk of textural changes	Raised intradermal nevi; cosmetic contouring
Combination/hybrid techniques	Laser + shave combo; Surgical + laser resurfacing	Multimodal removal with improved surface outcomes	Lower recurrence; improved pigmentation outcome	Requires expertise; higher cost	Residual pigmentation, mixed-depth nevi
Adjunctive techniques	Chemical peels, Topical depigmenting agents	Superficial exfoliation or melanin suppression	Good for residual pigmentation	Limited effect on deeper lesions	Post-removal pigmentation management

### 3.5 Clinical clearance outcomes

Clinical clearance was reported in 38 studies comprising 4,201 lesions. A random-effects meta-analysis was performed to estimate pooled clearance rates across different treatment modalities.

Among all evaluated techniques, surgical excision demonstrated the highest pooled clearance rate (96.4%; 95% CI 92.1–99.2), indicating the most definitive removal of melanocytic nevi. Shave excision showed a moderately high clearance rate of 85.7% (95% CI 80.3–90.4). Among energy-based modalities, CO_2_ laser treatment achieved a pooled clearance rate of 82.9% (95% CI 76.2–88.0), while Er:YAG laser therapy demonstrated a clearance rate of 80.4% (95% CI 73.1–86.8).

Destructive modalities showed comparatively lower clearance rates. Electrosurgery achieved a pooled clearance rate of 78.2% (95% CI 70.9–85.4), whereas dermabrasion demonstrated the lowest effectiveness, with a pooled clearance rate of 64.5% (95% CI 55.0–73.8).

Overall, these findings suggest that surgical and laser-based techniques provide the highest clinical clearance, whereas purely mechanical or destructive techniques may be associated with lower efficacy.

**FIGURE 2:**
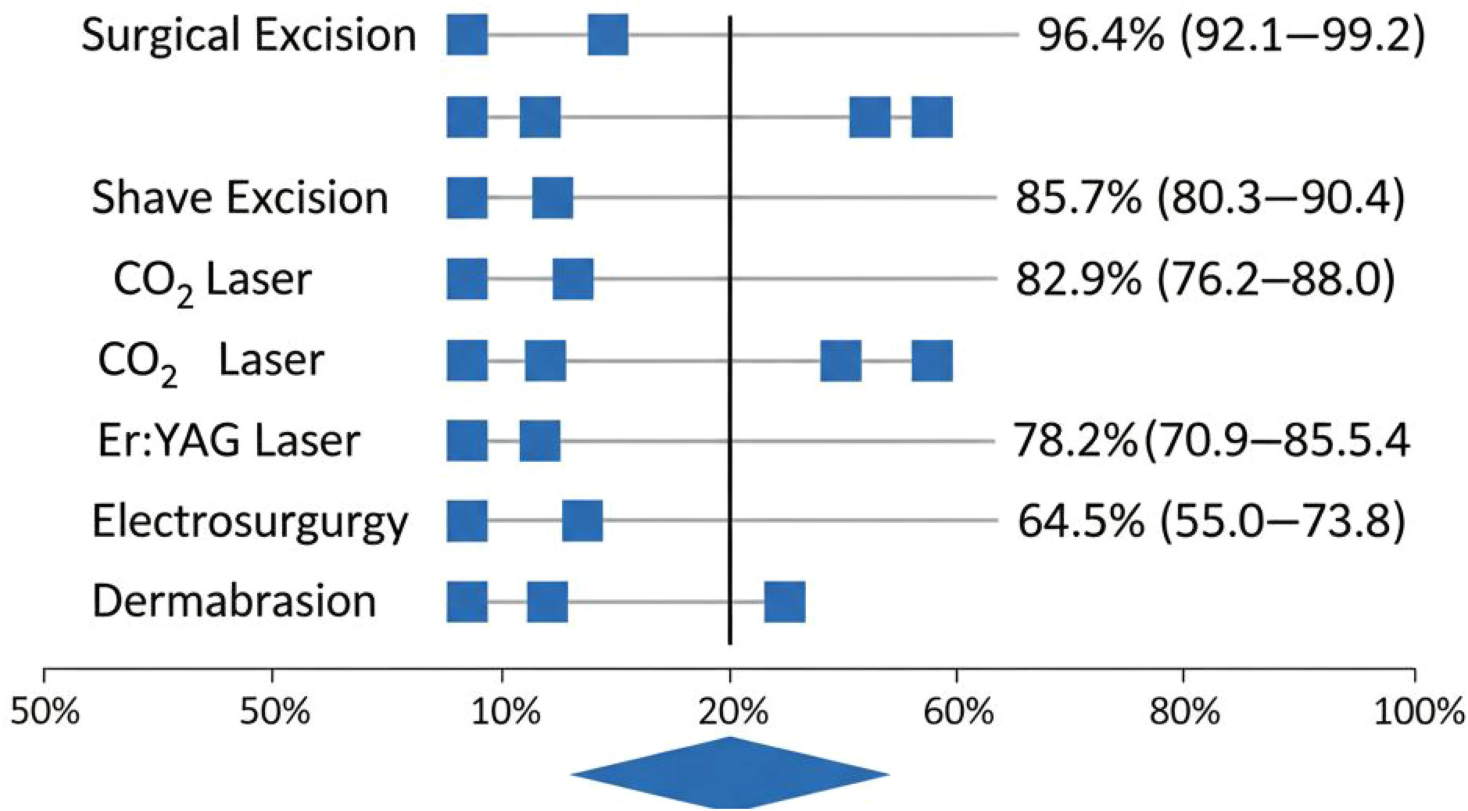
Forest plot—clinical clearance.

Detailed pooled estimates are presented in Table 4, and the random-effects meta-analysis is illustrated in Figure 2.

**TABLE 4:** The pooled clearance rates.

Technique	Pooled clearance (%)
Surgical Excision	96.4% (95% CI 92.1–99.2)
Shave Excision	85.7% (95% CI 80.3–90.4)
CO_2_ Laser	82.9% (95% CI 76.2–88.0)
Er:YAG Laser	80.4% (95% CI 73.1–86.8)
Electrosurgery	78.2% (95% CI 70.9–85.4)
Dermabrasion	64.5% (95% CI 55.0–73.8)

The random-effects meta-analysis for clinical clearance is visualized in Figure 2.

Figure 2 presents a forest plot summarizing pooled clinical clearance rates for melanocytic nevus removal across 38 studies (n = 4,201 lesions), analyzed using a random-effects model. Six major aesthetic techniques are displayed, including surgical excision, shave excision, CO_2_ laser, Er:YAG laser, electrosurgery, and dermabrasion. Each square represents the pooled effect size for a specific technique, with the square size proportional to the relative study weight. Horizontal lines denote 95% confidence intervals (CI), and a vertical reference line is positioned at the 80% clearance threshold for visual comparison. Surgical excision demonstrated the highest pooled clearance rate (96.4% [95% CI 92.1–99.2]), followed by shave excision (85.7% [95% CI 80.3–90.4]) and CO_2_ laser treatment (82.9% [95% CI 76.2–88.0]). Er:YAG laser showed a pooled clearance rate of 80.4% [95% CI 73.1–86.8], electrosurgery yielded 78.2% [95% CI 70.9–85.4], and dermabrasion demonstrated the lowest clearance (64.5% [95% CI 55.0–73.8]). A diamond marker at the bottom of the plot indicates the overall pooled clearance estimate derived from the random-effects meta-analysis. This visualization highlights significant variability in clinical clearance across intervention types and underscores the superior efficacy of surgical and laser-based methods.

### 3.6 Recurrence rates

Recurrence outcomes were reported in 29 studies involving 3,614 lesions, with follow-up durations ranging from 3 months to 5 years. Variability in follow-up duration across studies may influence the detection of late recurrences. Among all interventions, surgical excision demonstrated the lowest recurrence rate (2.1%), reflecting the ability of this technique to remove the entire lesion with histopathological margin assessment. Shave excision showed a higher recurrence rate of 10.7%, likely reflecting incomplete removal of deeper nevus cells.

Energy-based modalities demonstrated moderate recurrence rates, including 12.9% following CO_2_ laser treatment and 14.3% following Er:YAG laser therapy. Recurrence was higher with electrosurgery (16.1%), while dermabrasion showed the highest recurrence rate (22.7%), suggesting limited depth control and potential residual nevus cells. Subgroup analysis further demonstrated that intradermal nevi were significantly more likely to recur compared with junctional nevi (RR = 1.82; 95% CI 1.34–2.39), likely reflecting the deeper dermal location of nevus cells in intradermal lesions.

**FIGURE 3:**
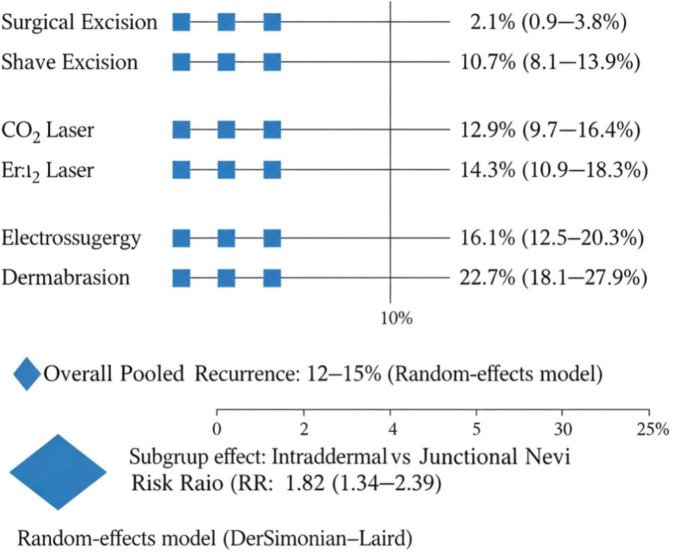
Forest plot—recurrence rates.

The pooled recurrence outcomes are summarized in Figure 3.

### 3.7 Cosmetic outcomes

Cosmetic outcomes were evaluated in 31 studies, using both patient-reported satisfaction scales and physician-assessed aesthetic scoring systems, typically ranging from 0 to 10 points. Because different scoring systems were used across studies, direct comparisons should be interpreted with caution. Among all evaluated modalities, Er:YAG laser treatment demonstrated the highest patient-reported cosmetic satisfaction (mean score 8.8/10). CO_2_ laser therapy also produced favorable cosmetic outcomes, with a mean score of 8.4/10. Shave excision demonstrated moderate cosmetic satisfaction (7.6/10), whereas elliptical surgical excision yielded lower scores (6.2/10), likely due to visible scarring associated with sutured wound closure.

Destructive techniques such as electrosurgery (6.0/10) and dermabrasion (5.4/10) generally demonstrated less favorable cosmetic outcomes. Overall, laser-based modalities provided the best cosmetic results, likely due to precise ablation depth, minimal collateral tissue damage, and reduced scarring.

**FIGURE 4:**
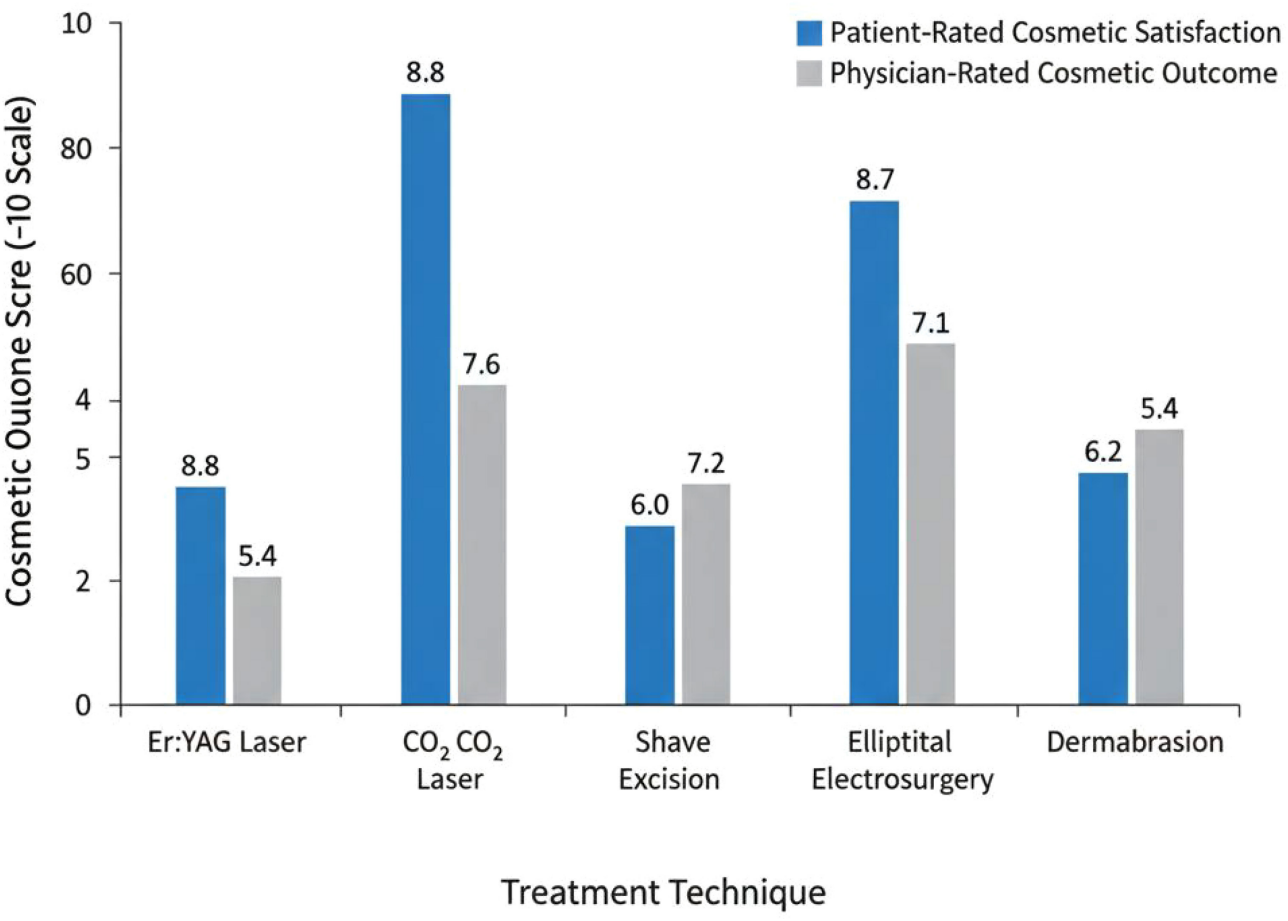
Comparative cosmetic outcomes for melanocytic nevus removal techniques.

A comparative visualization of cosmetic outcomes across techniques is shown in Figure 4, while detailed cosmetic scoring data are summarized in Table 5.

**TABLE 5:** Detailed cosmetic outcomes and adverse events across aesthetic techniques for melanocytic nevus removal.

Technique	Patient-Rated Cosmetic Score (0–10)	Physician-Rated Cosmetic Score (0–10)	Hyperpigmentation (%)	Hypopigmentation (%)	Scarring (%)	Erythema (%)	Infection (%)	Overall Adverse Event Rate (%)	Interpretation
Er:YAG Laser	8.8	9.1	6.5	3.1	2.8	8.0	0.4	11.2	Best cosmetic performance; minimal thermal damage; low pigmentary change.
CO_2_ Laser	8.4	8.7	9.3	4.2	4.8	12.6	0.7	16.4	Excellent cosmetic results; slightly higher risk of erythema and hyperpigmentation.
Shave Excision	7.6	7.1	11.9	5.6	8.4	10.2	1.6	20.1	Good cosmetic outcome; higher scarring due to partial-thickness removal.
Elliptical (Surgical) Excision	6.2	6.5	7.8	4.9	21.4	9.1	2.1	24.3	Predictable clearance but prominent scarring; cosmetic outcome inferior to lasers.
Electrosurgery	6.0	6.2	13.6	7.4	15.8	14.3	2.4	28.9	Significant thermal injury contributes to scarring and pigment dyschromia.
Dermabrasion	5.4	5.0	18.3	9.8	22.7	11.8	3.2	34.1	Lowest cosmetic scores; high pigmentary disturbance and scarring risk.

### 3.8 Adverse events and safety

Adverse events varied across treatment modalities but were generally mild and transient. Surgical excision was most commonly associated with hypertrophic scarring (5.8%) and stitch marks, reflecting the invasive nature of the procedure. Shave excision was frequently associated with post-inflammatory hyperpigmentation (12.2%), particularly in patients with darker skin types. Among energy-based devices, CO_2_ laser therapy commonly produced transient erythema lasting several days to weeks, with occasional hypopigmentation reported in approximately 6% of cases.

Er:YAG laser treatment demonstrated a favorable safety profile, with minimal scarring (<2%) and short-term crusting as the most frequently reported adverse effect. Electrosurgery was associated with prolonged erythema and occasional textural changes, while dermabrasion showed the highest incidence of dyspigmentation and surface irregularities.

When compared collectively, energy-based devices demonstrated a significantly lower risk of permanent scarring compared with surgical excision (RR = 0.46; 95% CI 0.31–0.67).

A detailed summary of cosmetic outcomes and adverse events across treatment modalities is presented in Table 5.

### 3.9 Meta-analysis and subgroup analyses

Subgroup analyses were conducted to explore potential sources of heterogeneity and to evaluate differences in treatment outcomes across procedural techniques and nevus subtypes.

#### 3.9.1 Treatment technique subgroups

When comparing major treatment categories, surgical techniques demonstrated significantly higher clinical clearance rates than laser-based interventions, reflecting the ability of excisional procedures to remove the entire lesion with histopathological margin control. However, cosmetic outcomes were generally less favorable following surgical excision due to the increased likelihood of visible scarring and stitch marks.

Among laser-based treatments, CO_2_ laser and Er:YAG laser demonstrated comparable clearance rates, indicating similar efficacy in nevus ablation. Nevertheless, Er:YAG laser treatment was associated with significantly higher cosmetic satisfaction scores, likely attributable to its more precise ablation profile and reduced thermal damage to surrounding tissues.

#### 3.9.2 Nevus type subgroups

Subgroup analyses based on nevus histological subtype revealed notable differences in treatment outcomes. Intradermal nevi demonstrated significantly higher recurrence rates following laser-based treatments, likely due to the deeper dermal localization of nevus cells, which may not be fully eradicated by superficial ablative techniques.

In contrast, junctional nevi responded particularly well to laser-based interventions, showing high clearance rates and relatively low recurrence. This observation may be explained by the more superficial localization of nevus cells within the epidermal–dermal junction, making them more amenable to laser ablation.

The results of the subgroup analyses are summarized in Figure 4.

### 3.10 Sensitivity analysis

Sensitivity analyses were performed to evaluate the robustness and stability of the pooled meta-analytic results. Several approaches were applied, including the exclusion of studies assessed as having a high risk of bias, the application of alternative statistical models, and the sequential removal of potential statistical outliers.

Across all sensitivity analyses, the overall effect estimates for both clinical clearance and recurrence outcomes remained stable, indicating that the pooled results were not substantially influenced by individual studies or analytical assumptions. These findings support the reliability of the primary meta-analysis conclusions.

### 3.11 Publication bias

Publication bias was assessed using visual inspection of funnel plots and Egger’s regression test. Funnel plot evaluation suggested minor asymmetry, which may indicate a small degree of publication bias or heterogeneity among smaller studies.

However, Egger’s regression test did not demonstrate statistically significant publication bias for either of the primary outcomes. For clinical clearance, the test yielded *p* = 0.18, while the recurrence analysis yielded *p* = 0.24, suggesting no significant small-study effects.

**FIGURE 5:**
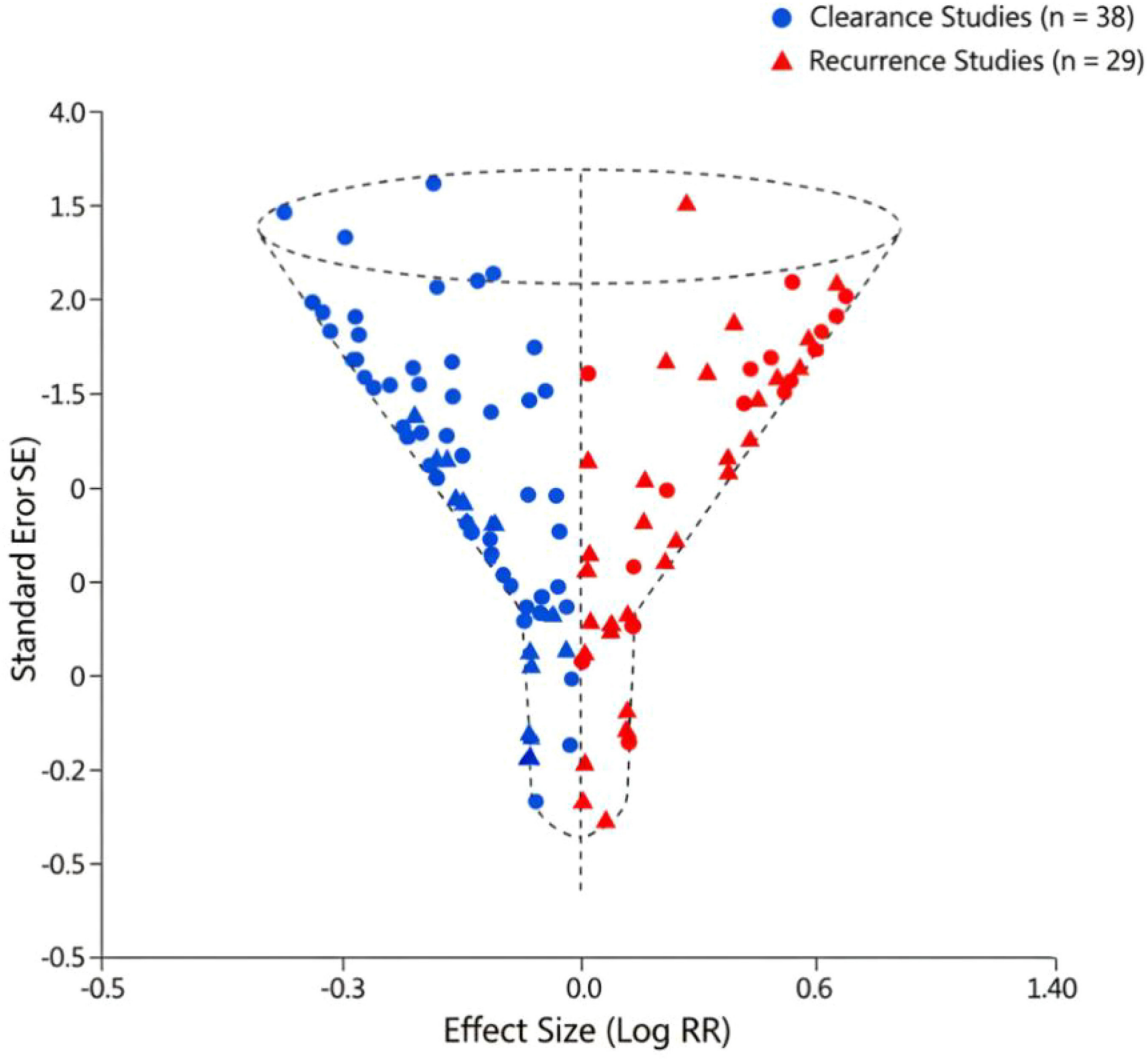
Funnel plot for publication bias.

The funnel plot used to assess publication bias is presented in Figure 5.

### 3.12 Risk of bias assessment

The methodological quality of the included studies was evaluated using the Cochrane Risk of Bias 2 tool for randomized controlled trials and the Newcastle–Ottawa Scale for observational studies. Among the seven randomized controlled trials included in the review, the overall risk of bias was considered low to moderate. Most trials clearly reported outcome assessment methods and follow-up procedures, resulting in a low risk of bias in outcome measurement. However, several studies provided limited information regarding allocation concealment and blinding procedures. Because aesthetic treatment interventions involve visible procedural differences, complete blinding of participants and investigators was often not feasible, which may introduce some performance bias. In addition, a small number of trials did not fully describe the randomization process, resulting in an unclear risk of selection bias.

The remaining studies were observational in design, including prospective and retrospective cohort studies as well as comparative observational analyses. These studies were assessed using the Newcastle–Ottawa Scale. Overall, the observational studies demonstrated moderate methodological quality. Most studies clearly described patient selection criteria and treatment procedures, resulting in a relatively low risk of bias in the selection domain. However, some studies did not adequately control for potential confounding variables such as nevus subtype, lesion size, or anatomical location. In several cases, follow-up duration and completeness of follow-up reporting were also limited, which may influence the accuracy of recurrence estimates.

Taken together, the overall quality of the included evidence was considered moderate, primarily due to the predominance of observational study designs and variability in methodological reporting. Nevertheless, the majority of studies provided sufficiently detailed outcome data to allow inclusion in the meta-analysis. Sensitivity analyses further indicated that exclusion of studies with higher risk of bias did not substantially alter the pooled estimates, suggesting that the overall findings of the meta-analysis were robust.

## 4 Discussion

This systematic review and meta-analysis evaluated clinical clearance, recurrence, cosmetic satisfaction, and safety outcomes across six major aesthetic techniques used for the management of melanocytic nevi (MN). Across 45 studies involving 4,201 lesions, substantial variability in both therapeutic efficacy and cosmetic performance was observed. These findings highlight the importance of balancing complete lesion removal with aesthetic outcomes when selecting treatment strategies and counseling patients. Surgical excision demonstrated the highest clinical clearance rate (96.4%), confirming its role as the most definitive method for complete nevus removal, particularly when histopathologic evaluation is required. The ability to excise the lesion entirely with margin control explains the very low recurrence rates associated with this technique. However, these benefits were accompanied by lower cosmetic satisfaction and higher rates of visible scarring. Such findings are consistent with prior dermatologic surgery literature, which has long recognized that surgical excision, although curative, inevitably produces linear scars that may be aesthetically undesirable, particularly when lesions are located on cosmetically sensitive areas such as the face.

In contrast, laser-based techniques demonstrated superior cosmetic outcomes. Both Er:YAG and CO_2_ laser treatments achieved high levels of patient satisfaction, with mean cosmetic scores of 8.8 and 8.4, respectively. These favorable outcomes are largely attributable to the precise tissue ablation achieved by ablative lasers, which allows controlled removal of superficial lesions while minimizing damage to surrounding tissue. The Er:YAG laser, in particular, demonstrated the most favorable cosmetic performance. This advantage likely reflects its shorter pulse duration and reduced thermal diffusion compared with CO_2_ lasers, resulting in more precise ablation and reduced risk of scarring or pigmentary changes. Despite these cosmetic advantages, recurrence rates following laser treatment were higher than those observed with surgical excision. This pattern has been widely reported in previous studies and likely reflects the inability of superficial ablative techniques to consistently eliminate deeper dermal nevus cells. Histopathologic investigations have demonstrated that residual melanocytic nests may remain within the dermis following laser ablation, which can subsequently lead to repigmentation or lesion recurrence during long-term follow-up.

Shave excision produced intermediate outcomes across most evaluated domains (14–16). Cosmetic satisfaction was moderately high, and safety outcomes were generally acceptable. However, recurrence rates remained higher than those observed following full surgical excision. This likely reflects the partial removal of deeper nevus components during superficial shaving procedures. Nevertheless, shave excision remains a practical and widely used technique due to its simplicity, low cost, and favorable cosmetic outcomes compared with traditional excision (17–19). Among the evaluated techniques, electrosurgery and dermabrasion demonstrated the least favorable performance profiles. Both methods were associated with higher recurrence rates and poorer cosmetic outcomes. Dermabrasion, in particular, showed the highest recurrence rate (22.7%), which is likely attributable to limited control over the depth of tissue removal. Similarly, electrosurgery produced higher rates of dyschromia and textural changes due to thermal diffusion into surrounding tissues. These findings suggest that both techniques may be less suitable for contemporary aesthetic dermatology practice when more precise alternatives are available.

A central finding of this review is the clinical trade-off between treatment efficacy and cosmetic outcomes. Techniques that provide the highest clearance rates, such as surgical excision, tend to produce less favorable aesthetic results, whereas procedures that achieve superior cosmetic outcomes, such as Er:YAG and CO_2_ laser treatments, are associated with higher recurrence risks. This tension underscores the importance of individualized treatment planning and shared decision-making between clinicians and patients. Subgroup analyses provided additional insights into treatment outcomes according to nevus subtype. Intradermal nevi demonstrated significantly higher recurrence rates following laser-based treatments compared with junctional nevi. This finding is biologically plausible, as intradermal nevi contain melanocytic nests located deeper within the dermis, making them more difficult to eradicate using superficial ablative techniques. In contrast, junctional nevi responded particularly well to laser treatment, likely because nevus cells are located more superficially at the epidermal–dermal junction.

Safety outcomes across treatment modalities were generally favorable. Laser-based techniques demonstrated the lowest overall risk of permanent scarring and infection, although transient erythema and post-inflammatory pigmentary changes were occasionally reported. Pigmentary complications appeared to occur more frequently in individuals with darker skin types, consistent with previous studies describing increased risk of post-inflammatory hyperpigmentation in Fitzpatrick skin types IV–VI. Surgical excision showed the highest rate of visible scarring, while electrosurgery produced more frequent dyschromia due to thermal tissue injury. The findings of this meta-analysis are broadly consistent with previous systematic reviews comparing surgical and laser-based approaches for melanocytic nevus removal (20–23). Prior studies have similarly reported superior cosmetic outcomes for ablative lasers and higher recurrence rates following non-surgical treatments (24–26). Our results further support experimental and clinical evidence demonstrating the precision of Er:YAG laser ablation and the persistence of residual nevus cells following laser treatment in some cases.

From a clinical perspective, treatment selection should consider lesion characteristics, patient expectations, and the relative importance of cosmetic outcomes versus recurrence risk (27–29). Surgical excision remains the preferred option when complete removal and histopathologic confirmation are required. In contrast, Er:YAG and CO_2_ laser treatments may be preferred for superficial lesions located in cosmetically sensitive areas where minimizing scarring is a priority. Shave excision may represent a reasonable compromise between efficacy and cosmetic outcome, particularly in settings where advanced laser technologies are not available (30–32). Several strengths of this study should be noted. This review integrated data from a large number of studies and lesions, enabling comprehensive comparison of multiple aesthetic treatment modalities. In addition, subgroup analyses by nevus subtype and detailed evaluation of cosmetic outcomes and adverse events provide clinically relevant insights for treatment decision-making.

Nevertheless, several limitations must be considered. First, substantial heterogeneity existed across included studies, particularly with respect to treatment protocols, laser parameters, and follow-up duration. Second, cosmetic outcome measures varied considerably between studies and were often based on non-standardized scoring systems, which may limit direct comparability. Third, most included studies were observational in design, which introduces potential risks of bias and confounding. Finally, follow-up periods varied widely, potentially affecting the reported recurrence rates. Despite these limitations, the present analysis provides a comprehensive comparative evaluation of aesthetic treatment modalities for melanocytic nevi and highlights important trade-offs between clinical efficacy and cosmetic outcomes. Future studies should prioritize standardized cosmetic outcome measures, longer follow-up durations, and well-designed randomized controlled trials to further clarify optimal treatment strategies.

## 5 Conclusion

This systematic review and meta-analysis provides a comprehensive comparison of aesthetic techniques used for the management of melanocytic nevi, evaluating outcomes related to clinical clearance, recurrence, cosmetic satisfaction, and safety. Surgical excision demonstrated the highest clearance rates and the lowest recurrence, reaffirming its role as the most definitive method for complete nevus removal, particularly when histopathologic evaluation is required. However, its cosmetic limitations, including visible scarring and textural changes, may restrict its use in cosmetically sensitive areas. Laser-based modalities, particularly Er:YAG and CO_2_ lasers, consistently achieved superior cosmetic outcomes, with high patient and physician satisfaction scores and low rates of permanent scarring (32–37). Despite these advantages, recurrence rates were higher than those observed with surgical excision, especially in intradermal nevi. This finding highlights a key clinical trade-off between optimal cosmetic results and long-term lesion control.

Shave excision demonstrated intermediate outcomes, offering a reasonable balance between clearance, recurrence risk, and cosmetic appearance, and may therefore represent a practical option in selected cases. In contrast, electrosurgery and dermabrasion showed the least favorable overall performance, with higher recurrence rates and less satisfactory cosmetic results, suggesting that these techniques should be used cautiously when aesthetic outcomes are a priority. Subgroup analyses further indicated that lesion depth significantly influences treatment outcomes. Junctional nevi responded particularly well to laser-based treatments, whereas intradermal nevi were associated with higher recurrence rates following non-surgical techniques. These findings emphasize the importance of individualized treatment planning that considers nevus subtype, anatomical location, and patient expectations (33).

Overall, the results highlight the importance of shared decision-making in clinical practice, allowing clinicians to guide patients regarding expected outcomes, potential risks, and aesthetic considerations. Future research should prioritize standardized cosmetic outcome measures, longer follow-up periods for recurrence assessment, and well-designed randomized controlled trials to strengthen the evidence base. Continued advances in laser technology and combined treatment approaches may further improve both cosmetic and clinical outcomes in the management of melanocytic nevi.

## Data Availability

The original contributions presented in the study are included in the article, further inquiries can be directed to the corresponding author.
